# New Iterative Method for Fractional Gas Dynamics and Coupled Burger's Equations

**DOI:** 10.1155/2015/153124

**Published:** 2015-03-26

**Authors:** Mohamed S. Al-luhaibi

**Affiliations:** Department of Mathematics, Faculty of Science, Kirkuk University, Kirkuk, Iraq

## Abstract

This paper presents the approximate analytical solutions to solve the nonlinear gas dynamics and coupled Burger's equations with fractional time derivative. By using initial values, the explicit solutions of the equations are solved by using a reliable algorithm. Numerical results show that the new iterative method is easy to implement and accurate when applied to time-fractional partial differential equations.

## 1. Introduction

Fractional calculus is a field of applied mathematics that deals with derivatives and integrals of arbitrary orders. During the last decade, fractional calculus has found applications in numerous seemingly diverse fields of science and engineering. Fractional differential equations are increasingly used to model problems in fluid mechanics, acoustics, biology, electromagnetism, diffusion, signal processing, and many other physical processes [1–15].

There exists a wide class of literature dealing with problems of approximate solutionsto fractional differential equations with various different methodologies, called perturbation methods. The perturbation methods have some limitations; for example, the approximate solution involves series or small parameters which pose difficulty since the majority of nonlinear problems have no small parameters at all. Although appropriate choices of small parameters sometimes lead to ideal solution, in most of the cases, unsuitable choices lead to serious effects in the solutions. Therefore, an analytical method is welcome which does not require a small parameter in the equation modeling the phenomenon. Also, that analytical method is welcome in which there is no need to calculate Adomian polynomials which require so much computational time for higher-order approximations, to calculate Lagrange multiplier value which requires the variational theory to calculate it or equating the terms of like powers of the embedding parameter* p* [16–18].

In this paper we use the new iterative method to solve the nonlinear gas dynamics and coupled Burger's equations with fractional time derivative; this method was proposed first by Daftardar-Gejji and Jafari [19] and has proven useful for solving a variety of nonlinear equations.

## 2. Preliminaries and Notations

In this section, we mention the following basic definitions of fractional calculus which are used further in the present work.

**Definition 1 .**

The Riemann-Liouville fractional integral operator of order *α* > 0, of a function *f*(*t*) ∈ *C*_*μ*_ and *μ* ≥ −1, is defined as [1]

(1) $$\begin{array}{c} I^{\alpha }f\left(t\right)=\frac{1}{\Gamma \left(\alpha \right)}\overset{t}{\underset{0}{\int }}{\left(t-\tau \right)}^{\alpha -1}f\left(\tau \right)d\tau ,\;\alpha >0,\\ I^{0}f\left(t\right)=f\left(t\right). \end{array}$$

For the Riemann-Liouville fractional integral, we have

(2) $$\begin{array}{c} I^{\alpha }t^{v}=\frac{\Gamma \left(v+1\right)}{\Gamma \left(v+1+\alpha \right)}t^{v+\alpha }. \end{array}$$

**Definition 2 .**

The fractional derivative of *f*(*t*) in the Caputo sense is defined as [5]

(3) Dtαft=Itm−αDmft=1Γm−α∫0tt−τm−α−1fmτdτ,hhhhhhhhhhhhm−1<α≤m, t>0.

From properties of *D*_*t*_^*α*^, it is important to note that

(4) $$\begin{array}{c} D_{t}^{\alpha }t^{v}=\frac{\Gamma \left(v+1\right)}{\Gamma \left(v+1-\alpha \right)}t^{v-\alpha }. \end{array}$$

For the Riemann-Liouville fractional integral and Caputo fractional derivative, we have the following relation:

(5) $$\begin{array}{c} I_{t}^{\alpha }D_{t}^{\alpha }f\left(t\right)=f\left(t\right)-\overset{m-1}{\underset{k=0}{\sum }}f^{k}\left(0_{+}\right)\frac{t^{k}}{k!}. \end{array}$$

## 3. Basic Idea of New Iterative Method

To describe the idea of the NIM, consider the following general functional equation [19–22]:

(6) $$\begin{array}{c} u\left(x\right)=f\left(x\right)+N\left(u\left(x\right)\right), \end{array}$$

where *N* is a nonlinear operator from a Banach space *B* → *B* and *f* is a known function. We are looking for a solution *u* of (6) having the series form

(7) $$\begin{array}{c} u\left(x\right)=\overset{\infty }{\underset{i=0}{\sum }}u_{i}\left(x\right). \end{array}$$

The nonlinear operator *N* can be decomposed as follows:

(8) $$\begin{array}{c} N\left(\overset{\infty }{\underset{i=0}{\sum }}u_{i}\right)=N\left(u_{0}\right)+\overset{\infty }{\underset{i=1}{\sum }}\left\{N\left(\overset{i}{\underset{j=0}{\sum }}u_{j}\right)-N\left(\overset{i-1}{\underset{j=0}{\sum }}u_{j}\right)\right\}. \end{array}$$

From (7) and (8), (6) is equivalent to

(9) $$\begin{array}{c} \overset{\infty }{\underset{i=0}{\sum }}u_{i}=f+N\left(u_{0}\right)+\overset{\infty }{\underset{i=1}{\sum }}\left\{N\left(\overset{i}{\underset{j=0}{\sum }}u_{j}\right)-N\left(\overset{i-1}{\underset{j=0}{\sum }}u_{j}\right)\right\}. \end{array}$$

We define the recurrence relation:

(10a) $$\begin{array}{c} u_{0}=f, \end{array}$$

(10b) $$\begin{array}{c} u_{1}=N\left(u_{0}\right), \end{array}$$

(10c) un+1=Nu0+u1+⋯+un−Nu0+u1+⋯+un−1,hhhhhhhhhhhhhhhhhhhhhhhhhhhh  n=1,2,3,….

Then

(11) u1+⋯+un+1=Nu0+u1+⋯+un,hhhhhhhhhn=1,2,3,…,u=∑i=0∞ui=f+N∑i=0∞ui.

If *N* is a contraction, that is,

(12) $$\begin{array}{c} \left\|N\left(x\right)-N\left(y\right)\right\|\leq k\left\|x-y\right\|,\;0<k<1, \end{array}$$

then

(13) un+1=Nu0+u1+⋯+un−Nu0+u1+⋯+un−1≤kun≤⋯≤knu0 n=0,1,2,…,

and the series ∑_*i*=0_^*∞*^ *u*_*i*_ absolutely and uniformly converges to a solution of (6) [23], which is unique, in view of the Banach fixed point theorem [24]. The *k*-term approximate solution of (6) is given by ∑_*i*=0_^*k*−1^ *u*_*i*_.

### 3.1. Reliable Algorithm of New Iterative Method for Solving the Linear and Nonlinear Partial Differential Equations

After the above presentation of the NIM, we introduce a reliable algorithm for solving nonlinear PDEs using the NIM. Consider the following nonlinear PDE of arbitrary order:

(14a) Dtαux,t=Au,∂u+Bx,t,hhhhhm−1<α≤m, m∈N

with the initial conditions

(14b) $$\begin{array}{c} \frac{\partial^{k}}{\partial t^{k}}u\left(x,0\right)=h_{k}\left(x\right),\;k=0,1,2,\ldots ,m-1, \end{array}$$

where *A* is a nonlinear function of *u* and ∂*u* (partial derivatives of *u* with respect to *x* and *t*) and *B* is the source function. In view of the integral operators, the initial value problem (14a) and (14b) is equivalent to the following integral equation:

(15) $$\begin{array}{c} u\left(x,t\right)=\overset{m-1}{\underset{k=0}{\sum }}h_{k}\left(x\right)\frac{t^{k}}{k!}+I_{t}^{\alpha }B\left(x,t\right)+I_{t}^{\alpha }A=f+N\left(u\right), \end{array}$$

where

(16) $$\begin{array}{c} f=\overset{m-1}{\underset{k=0}{\sum }}h_{k}\left(x\right)\frac{t^{k}}{k!}+I_{t}^{\alpha }B\left(x,t\right), \end{array}$$

(17) $$\begin{array}{c} N\left(u\right)=I_{t}^{\alpha }A, \end{array}$$

where *I*_*t*_^*n*^ is an integral operator of *n* fold. We get the solution of (15) by employing the algorithms (10a), (10b), and (10c).

## 4. Applications

**Example 1 .**

Consider the following nonlinear time-fractional gas dynamics equation:

(18a) $$\begin{array}{c} D_{t}^{\alpha }u\left(x,t\right)+\frac{1}{2}{\left(u^{2}\right)}_{x}-u\left(1-u\right)=0,\;0<\alpha \leq 1, \end{array}$$

with the initial condition

(18b) $$\begin{array}{c} u\left(x,0\right)=e^{-x}. \end{array}$$

From (10a) and (16), we obtain

(19) $$\begin{array}{c} u_{0}\left(x,t\right)=e^{-x}. \end{array}$$

Therefore, from (15), the initial value problem (18a) and (18b) is equivalent to the following integral equation:

(20) $$\begin{array}{c} u\left(x,t\right)=e^{-x}-I_{t}^{\alpha }\left(\frac{1}{2}{\left(u^{2}\right)}_{x}-u\left(1-u\right)\right),\\ N\left(u\right)=-I_{t}^{\alpha }\left(\frac{1}{2}{\left(u^{2}\right)}_{x}-u\left(1-u\right)\right). \end{array}$$

Therefore, from (10a), (10b), and (10c), we can obtain easily the following first few components of the new iterative solution for (18a) and (18b):

(21) u0x,t=e−x,u1x,t=e−xtαΓ1+α,u2x,t=e−xt2αΓ1+2α,u3x,t=e−xt3αΓ1+3α,⋮ 

and so on. The *n*-order term approximate solution, in series form, is given by

(22) unx,t =e−x1+tαΓ1+α+t2αΓ1+2α+t3αΓ1+3α+⋯.

In the special case, *α* = 1, (22) becomes

(23) $$\begin{array}{c} u_{n}\left(x,t\right)=e^{-x}\left(1+t+\frac{t^{2}}{2!}+\frac{t^{3}}{3!}+\frac{t^{4}}{4!}+\cdots \right). \end{array}$$

**Figure 1:**
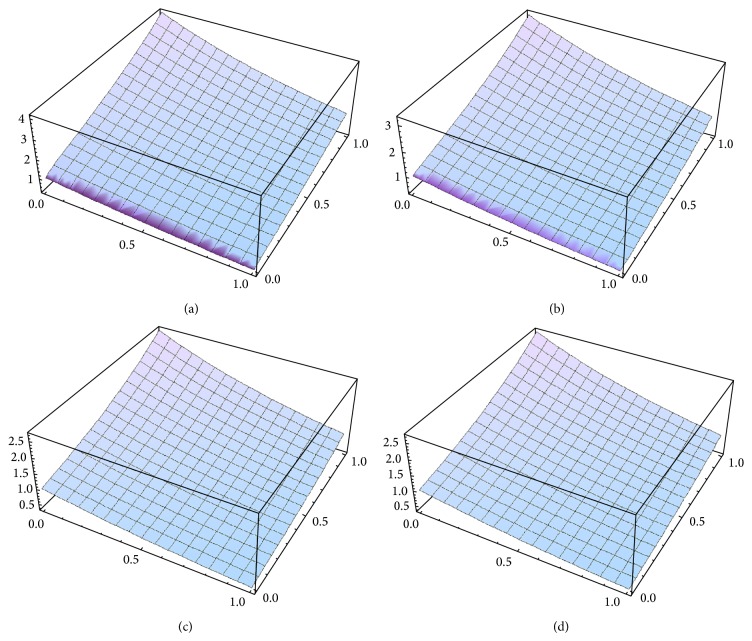
(a) 5-term approximate solution for (18a) and (18b), in case *x* : 0 → 1, *t* : 0 → 1, and *α* = 0.6. (b) 5-term approximate solution for (18a) and (18b), in case *x* : 0 → 1, *t* : 0 → 1, and *α* = 0.8. (c) 5-term approximate solution for (18a) and (18b), in case *x* : 0 → 1, *t* : 0 → 1, and *α* = 1. (d) Exact solution for (18a) and (18b), in case *x* : 0 → 1, *t* : 0 → 1, and *α* = 1.

In closed form, this gives

(24) $$\begin{array}{c} u_{n}\left(x,t\right)=\overset{\infty }{\underset{n=0}{\sum }}u_{n}\left(x,t\right)=e^{t-x}, \end{array}$$

which is the exact solution for (18a) and (18b) in the special case *α* = 1. The 5-order term approximate solution and the corresponding exact solution for (18a) and (18b) are plotted in Figure 1(a), for *α* = 0.6; in Figure 1(b), for *α* = 0.8; in Figure 1(c), for *α* = 1, and in Figure 1(d) the exact solution. It is remarkable to note that the surface of the approximate solution converges to the surface of the exact solution as *α* → 1. It is evident that the efficiency of the NIM can be dramatically enhanced by computing further terms of *u*(*x*, *t*).

**Example 2 .**

Consider the following time-fractional coupled Burgers equations in (1 + 1)-dimension:

(25a) $$\begin{array}{c} D_{t}^{\alpha }u\left(x,t\right)-u_{xx}-2uu_{x}+{\left(uv\right)}_{x}=0,\\ D_{t}^{\alpha }v\left(x,t\right)-v_{xx}-2uu_{x}+{\left(uv\right)}_{x}=0, \end{array}$$

with the initial conditions

(25b) $$\begin{array}{c} u\left(x,0\right)=e^{x},\;\;v\left(x,0\right)=e^{x}. \end{array}$$

From (10a) and (16), we obtain

(26) $$\begin{array}{c} u_{0}\left(x,t\right)=e^{x},\\ v_{0}\left(x,t\right)=e^{x}. \end{array}$$

Therefore, from (15), the initial value problems (25a) and (25b) are equivalent to the following integral equations:

(27) $$\begin{array}{c} u\left(x,t\right)=e^{x}+I_{t}^{\alpha }\left(u_{xx}+2uu_{x}-{\left(uv\right)}_{x}\right),\\ v\left(x,t\right)=e^{x}+I_{t}^{\alpha }\left(v_{xx}+2uu_{x}-{\left(uv\right)}_{x}\right),\\ N\left(u\right)=I_{t}^{\alpha }\left(u_{xx}+2uu_{x}-{\left(uv\right)}_{x}\right),\\ N\left(u\right)=I_{t}^{\alpha }\left(v_{xx}+2uu_{x}-{\left(uv\right)}_{x}\right). \end{array}$$

Therefore, from (10a), (10b), and (10c), we can obtain easily the following first few components of the new iterative solution for (25a) and (25b):

(28) $$\begin{array}{c} u_{0}\left(x,t\right)=e^{x},\\ v_{0}\left(x,t\right)=e^{x},\\ u_{1}\left(x,t\right)=e^{x}\left(\frac{t^{\alpha }}{\Gamma \left(1+\alpha \right)}\right),\\ v_{1}\left(x,t\right)=e^{x}\left(\frac{t^{\alpha }}{\Gamma \left(1+\alpha \right)}\right),\\ u_{2}\left(x,t\right)=e^{x}\left(\frac{t^{2\alpha }}{\Gamma \left(1+2\alpha \right)}\right),\\ v_{2}\left(x,t\right)=e^{x}\left(\frac{t^{2\alpha }}{\Gamma \left(1+2\alpha \right)}\right),\\ u_{3}\left(x,t\right)=e^{x}\left(\frac{t^{3\alpha }}{\Gamma \left(1+3\alpha \right)}\right),\\ v_{3}\left(x,t\right)=e^{x}\left(\frac{t^{3\alpha }}{\Gamma \left(1+3\alpha \right)}\right),\\ \vdots \end{array}$$

and so on. The *n*-order term approximate solution, in series form, is given by

(29) unx,t =ex1+tαΓ1+α+t2αΓ1+2α+t3αΓ1+3α+⋯,vnx,t =ex1+tαΓ1+α+t2αΓ1+2α+t3αΓ1+3α+⋯.

In the special case, *α* = 1, (29) becomes

(30) $$\begin{array}{c} u_{n}\left(x,t\right)=e^{-x}\left(1+t+\frac{t^{2}}{2!}+\frac{t^{3}}{3!}+\frac{t^{4}}{4!}+\cdots \right),\\ v_{n}\left(x,t\right)=e^{-x}\left(1+t+\frac{t^{2}}{2!}+\frac{t^{3}}{3!}+\frac{t^{4}}{4!}+\cdots \right). \end{array}$$

**Figure 2:**
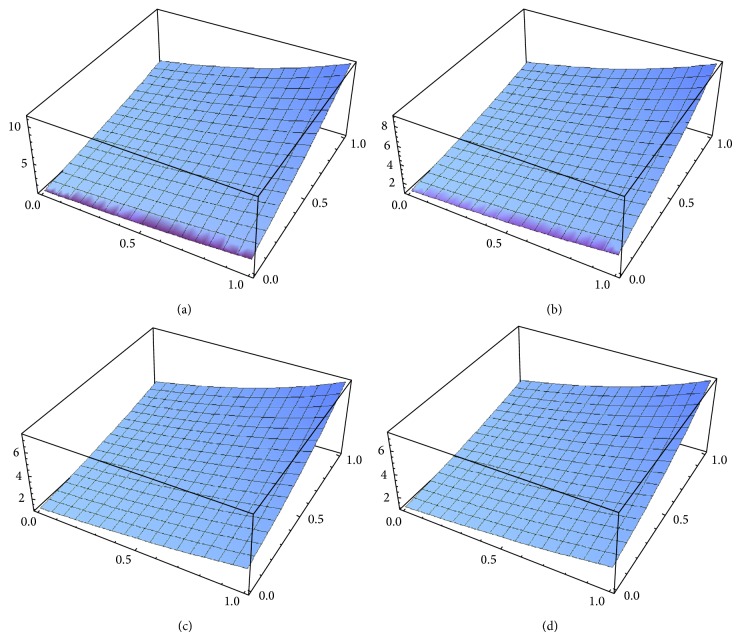
(a) 5-term approximate solution for *u*(*x*, *t*) and *v*(*x*, *t*), in case *x* : 0 → 1, *t* : 0 → 1, and *α* = 0.6. (b) 5-term approximate solution for *u*(*x*, *t*) and *v*(*x*, *t*), in case *x* : 0 → 1, *t* : 0 → 1, and *α* = 0.8. (c) 5-term approximate solution for *u*(*x*, *t*) and *v*(*x*, *t*), in case *x* : 0 → 1, *t* : 0 → 1, and *α* = 1. (d) Exact solution for *u*(*x*, *t*) and *v*(*x*, *t*), in case *x* : 0 → 1, *t* : 0 → 1, and *α* = 1.

In closed form, this gives

(31) $$\begin{array}{c} u_{n}\left(x,t\right)=\overset{\infty }{\underset{n=0}{\sum }}u_{n}\left(x,t\right)=e^{x+t},\\ v_{n}\left(x,t\right)=\overset{\infty }{\underset{n=0}{\sum }}v_{n}\left(x,t\right)=e^{x+t}, \end{array}$$

which is the exact solution for (25a) and (25b) in the special case *α* = 1. The 5-order term approximate solution and the corresponding exact solution for (25a) and (25b) are plotted in Figure 2(a), for *α* = 0.6; in Figure 2(b), for *α* = 0.8; in Figure 2(c), for *α* = 1; and in Figure 2(d), the exact solution. It is remarkable to note that the surface of the approximate solution converges to the surface of the exact solution as *α* → 1. It is evident that the efficiency of the NIM can be dramatically enhanced by computing further terms of *u*(*x*, *t*) and *v*(*x*, *t*).

**Example 3 .**

Consider the following time-fractional coupled Burgers equations in (2 + 1)-dimension:

(32a) Dtαux,y,t−∇2u−2u∇u+uvx+vuvy=0,Dtαux,y,t−∇2v−2v∇v+uvx+vuvy=0,hhhhhhhhhhhhhhhhhhhhhhhhhhhhhh0<α≤1,

with the initial conditions

(32b) $$\begin{array}{c} u\left(x,y,0\right)=e^{x+y},\;\;v\left(x,y,0\right)=e^{x+y}. \end{array}$$

From (10a) and (16), we obtain

(33) $$\begin{array}{c} u_{0}\left(x,y,t\right)=e^{x+y},\\ v_{0}\left(x,y,t\right)=e^{x+y}. \end{array}$$

Therefore, from (15), the initial value problems (32a) and (32b) are equivalent to the following integral equations:

(34) $$\begin{array}{c} u\left(x,y,t\right)=e^{x+y}+I_{t}^{\alpha }\left(\nabla^{2}u+2u\nabla u-{\left(uv\right)}_{x}-v{\left(uv\right)}_{y}\right),\\ v\left(x,y,t\right)=e^{x+y}+I_{t}^{\alpha }\left(\nabla^{2}u+2u\nabla u-{\left(uv\right)}_{x}-v{\left(uv\right)}_{y}\right),\\ N\left(u\right)=I_{t}^{\alpha }\left(\nabla^{2}u+2u\nabla u-{\left(uv\right)}_{x}-v{\left(uv\right)}_{y}\right),\\ N\left(u\right)=I_{t}^{\alpha }\left(\nabla^{2}u+2u\nabla u-{\left(uv\right)}_{x}-v{\left(uv\right)}_{y}\right). \end{array}$$

Therefore, from (10a), (10b), and (10c), we can obtain easily the following first few components of the new iterative solution for (32a) and (32b):

(35) $$\begin{array}{c} u_{0}\left(x,y,t\right)=e^{x+y},\\ v_{0}\left(x,y,t\right)=e^{x+y},\\ u_{1}\left(x,y,t\right)=e^{x+y}\left(\frac{2t^{\alpha }}{\Gamma \left(1+\alpha \right)}\right),\\ v_{1}\left(x,y,t\right)=e^{x+y}\left(\frac{2t^{\alpha }}{\Gamma \left(1+\alpha \right)}\right),\\ u_{2}\left(x,y,t\right)=e^{x+y}\left(\frac{{\left(2t^{\alpha }\right)}^{2}}{\Gamma \left(1+2\alpha \right)}\right),\\ v_{2}\left(x,y,t\right)=e^{x+y}\left(\frac{{\left(2t^{\alpha }\right)}^{2}}{\Gamma \left(1+2\alpha \right)}\right),\\ u_{3}\left(x,y,t\right)=e^{x+y}\left(\frac{{\left(2t^{\alpha }\right)}^{3}}{\Gamma \left(1+3\alpha \right)}\right),\\ v_{3}\left(x,y,t\right)=e^{x+y}\left(\frac{{\left(2t^{\alpha }\right)}^{3}}{\Gamma \left(1+3\alpha \right)}\right),\\ \vdots \end{array}$$

and so on. The *n*-order term approximate solution, in series form, is given by

(36) unx,y,t =ex+y1+tαΓ1+α+2tα2Γ1+2α+3tα2Γ1+3α+⋯,vnx,y,t =ex+y1+tαΓ1+α+2tα2Γ1+2α+3tα2Γ1+3α+⋯.

In the special case, *α* = 1, (36) becomes

(37) $$\begin{array}{c} u_{n}\left(x,y,t\right)=e^{x+y}\left(1+2t+\frac{{\left(2t\right)}^{2}}{2!}+\frac{{\left(3t\right)}^{2}}{3!}+\cdots \right),\\ v_{n}\left(x,y,t\right)=e^{x+y}\left(1+2t+\frac{{\left(2t\right)}^{2}}{2!}+\frac{{\left(3t\right)}^{2}}{3!}+\cdots \right). \end{array}$$

**Figure 3:**
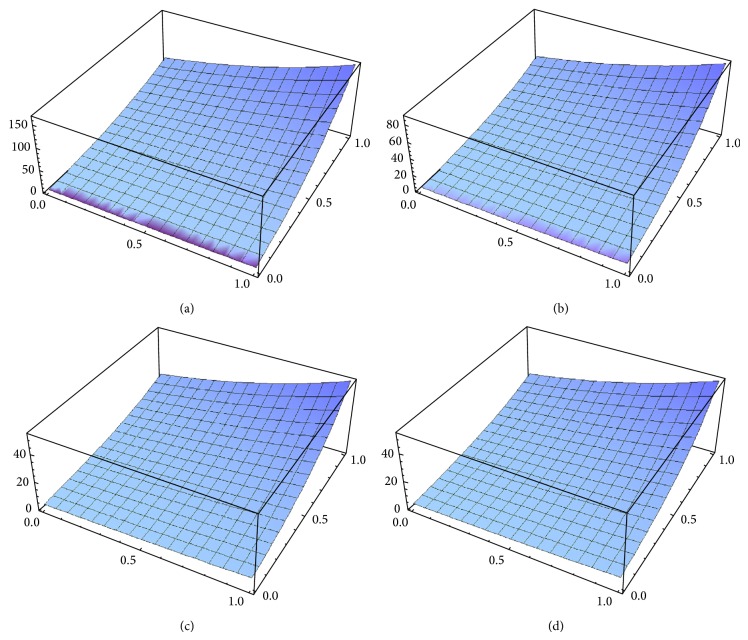
(a) 5-term approximate solution for *u*(*x*, *t*) and *v*(*x*, *t*), in case *y* = 1, *x* : 0 → 1, *t* : 0 → 1, and *α* = 0.6. (b) 5-term approximate solution for *u*(*x*, *t*) and *v*(*x*, *t*), in case *y* = 1, *x* : 0 → 1, *t* : 0 → 1, and *α* = 0.8. (c) 5-term approximate solution for *u*(*x*, *t*) and *v*(*x*, *t*), in case *y* = 1, *x* : 0 → 1, *t* : 0 → 1, and *α* = 1. (d) Exact solution for *u*(*x*, *t*) and *v*(*x*, *t*), in case *y* = 1, *x* : 0 → 1, *t* : 0 → 1, and *α* = 1.

In closed form, this gives

(38) $$\begin{array}{c} u_{n}\left(x,y,t\right)=\overset{\infty }{\underset{n=0}{\sum }}u_{n}\left(x,y,t\right)=e^{x+y+2t},\\ v_{n}\left(x,t\right)=\overset{\infty }{\underset{n=0}{\sum }}v_{n}\left(x,t\right)=e^{x+y+2t}, \end{array}$$

which is the exact solution for (32a) and (32b) in the special case *α* = 1. The 5-order term approximate solution and the corresponding exact solution for (32a) and (32b) are plotted in Figure 3(a), for *α* = 0.6; in Figure 3(b), for *α* = 0.8; in Figure 3(c), for *α* = 1; and in Figure 3(d), the exact solution. It is remarkable to note that the surface of the approximate solution converges to the surface of the exact solution as *α* → 1. It is evident that the efficiency of the NIM can be dramatically enhanced by computing further terms of *u*(*x*, *t*) and *v*(*x*, *t*).

## 5. Conclusion

In this work, the new iterative method (NIM) is successfully applied for solving nonlinear time-fractional gas dynamics equation and time-fractional coupled Burger's equations in (1 + 1)- and (2 + 1)-dimensions. The obtained results show that the surfaces of the approximate solutions are convergent to the surfaces of the corresponding exact solutions as *α* → 1. Therefore, this method is very powerful and efficient technique for solving nonlinear fractional differential equations arising in different fields of science. However, the NIM has more advantages over the other methods which are the following: (1) it solves the nonlinear problems without using Adomian polynomials, (2) it solves the problems without using Lagrange multiplier value, and (3) it solves the problems without using small parameters. In conclusion, the NIM may be considered as a nice refinement in existing numerical techniques and might find the wide applications.
